# Custom mentholation of commercial cigarettes for research purposes

**DOI:** 10.1016/j.toxrep.2014.10.009

**Published:** 2014-10-22

**Authors:** Ian C. MacGregor, Stephen B. Stanfill, Sydney M. Gordon, Douglas J. Turner, Jenny M. Butler, Elizabeth A. Hanft, Hyoshin Kim, Robyn R. Kroeger, Marielle C. Brinkman, Margaret E. Tefft, Pamela I. Clark, Stephanie S. Buehler

**Affiliations:** a Tobacco Exposure Research Laboratory, Battelle, Columbus, OH, United States; b Centers for Disease Control and Prevention, Atlanta, GA, United States; c Department of Behavioral and Community Health, School of Public Health, University of Maryland, College Park, MD, United States

**Keywords:** Menthol, Matched cigarettes, Nicotine, Cigarette tobacco, Tobacco control, Human exposure

## Abstract

In the U.S. menthol remains the sole permitted characterizing cigarette flavor additive in part because efforts to link menthol cigarette use to increased tobacco-related disease risk have been inconclusive. To perform definitive studies, cigarettes that differ only in menthol content are required, yet these are not commercially available. We prepared research cigarettes differing only in menthol content by deposition of l-menthol vapor directly onto commercial nonmenthol cigarettes, and developed a method to measure a cigarette's menthol and nicotine content. With our custom-mentholation technique we achieved the desired moderately high menthol content (as compared to commercial brands) of 6.7 ± 1.0 mg/g (*n* = 25) without perturbing the cigarettes’ nicotine content (17.7 ± 0.7 mg/g [*n* = 25]). We also characterized other pertinent attributes of our custom-mentholated cigarettes, including percent transmission of menthol and nicotine to mainstream smoke and the rate of loss of menthol over time during storage at room temperature. We are currently using this simple mentholation technique to investigate the differences in human exposure to selected chemicals in cigarette smoke due only to the presence of the added menthol. Our cigarettes will also aid in the elucidation of the effects of menthol on the toxicity of tobacco smoke.

## Highlights

• We developed a simple and repeatable technique to create research cigarettes that differ only in menthol content.
• We qualified a method by which the menthol and nicotine content of a cigarette can be quantified.
• We investigated the rate of loss of menthol from our custom-mentholated cigarettes over time during storage.
• We are currently using these custom-mentholated cigarettes in human exposure studies.
• Our custom-mentholated cigarettes will help to elucidate the effects of menthol on the toxicity of tobacco smoke.

## 1 Introduction

In the US menthol is the only characterizing flavor in cigarettes still permitted under the Family Smoking Prevention & Tobacco Control Act [1], but the law calls for research on the impact on public health of its continued use as a flavorant. Previous studies have demonstrated that menthol in tobacco smoke: changes brain chemistry and alters nicotine's addictive properties [2]; impacts biochemical processes such as the metabolism of nicotine [3], [4], [5]; and may cause smokers to inhale more deeply or hold their breath longer, thereby potentially causing greater exposure to the toxins in tobacco smoke [4]. In addition, menthol cigarettes are preferred by African Americans, and while African Americans smoke fewer cigarettes per day and tend to begin smoking later in life than do whites, African American males are at greater risk for smoking-related lung cancer, and their total smoking-related mortality from diseases associated with tobacco use is higher [6], [7]. Nonetheless, epidemiologic studies attempting to link menthol cigarette use to increased risk of tobacco-related disease have been inconclusive, largely because (1) such studies lack the power to measure a small difference in harm in the presence of the overwhelming harm associated with smoking any tobacco product, and (2) it is difficult to identify “menthol cigarette users” without error, particularly since most of the reported studies were not originally designed to address menthol in cigarettes [8], [9], [10], [11], [12], [13], [14], [15], [16].

Laboratory-based studies have also yielded mixed results because of compliance issues that require established menthol or nonmenthol cigarette smokers to use the opposite cigarette style for the extended periods necessary to compare classic measures of toxicity [7]. For example, when comparing biomarkers of exposure between menthol and nonmenthol smokers (e.g., cotinine, carbon monoxide [CO]), some studies showed decreased levels, some increased, and some no difference [4], [17], [18], [19], [20], [21], [22], [23]. The reason for this may be that commercial cigarettes are so highly engineered that there are many significant differences between menthol and nonmenthol cigarettes other than menthol levels. In earlier studies conducted using closely matched commercial menthol and nonmenthol brand pairs [24], [25], [26], we found increased exposures to 4-(methylnitrosamino)-1-(3-pyridyl)-1-butanone (NNK), a potent lung carcinogen [27]. We also measured greater exposures to smaller diameter particles in both mainstream and sidestream smoke from menthol cigarettes. However, despite the cigarettes used in these studies having matching smoke yields [28], we cannot attribute the increased exposures observed with the menthol cigarettes to the effects of menthol alone. To adequately study the effect of menthol in cigarettes, cigarettes that differ only in menthol content are needed.

The objective of this study was to generate matched menthol and nonmenthol cigarettes that differ only in menthol content to support our related human exposure studies designed to elucidate the differences, due to menthol, in smokers’ exposures to particles and to harmful and potentially harmful constituents (HPHCs; [29]) in mainstream smoke, differences that could help inform the effects of menthol on the toxicity of mainstream smoke. Note that we did not seek to mimic the mentholation process used by industry, nor to replicate the menthol content of a specific commercial cigarette. Rather, our goal was to produce cigarettes with a known amount of menthol at the upper end of the range of the levels reported for commercial brands so as to maximize the likelihood for measuring potential differences in our human exposure studies. To accomplish these goals we developed a technique to generate cigarettes at predefined and reproducible levels of menthol. We also developed and qualified a method to co-extract and measure both the menthol and nicotine content of the tobacco rod and cigarette filter, as it is important that the amount of menthol and nicotine in the custom-mentholated cigarettes be accurately characterized for our ongoing exposures studies.

This paper describes our custom mentholation procedure based on direct vapor deposition, the menthol and nicotine analysis method adopted, and the assessment of pertinent characteristics of our custom-mentholated cigarettes that serve to verify their similarity to their nonmentholated precursors. These characteristics included their menthol and nicotine content, the distribution of menthol and nicotine between the tobacco rod and filter, the transfer efficiency of both menthol and nicotine from the tobacco rod to mainstream smoke, and the rate of loss of menthol and nicotine from the stored cigarettes over time.

## 2 Methods

### 2.1 Extraction and analysis of menthol and nicotine

To evaluate the menthol and nicotine content of the unburned cigarettes, we separated each cigarette into rod (tobacco and paper) and filter, weighed them to the nearest 0.1 mg, and extracted and analyzed the rod and filter separately using a technique adapted from previously published work [30]. Extraction was performed using a solution of 0.8 mL isopropanol (Fisher), 20 mL methyl *tert*-butyl ether (MTBE; Sigma–Aldrich) containing a surrogate compound, quinoline (Sigma–Aldrich) at 100 μg/mL, and 2 mL of 2 N sodium hydroxide (Sigma–Aldrich). After agitation on an orbital shaker for 4 h at 160 rotations per minute (rpm), the resulting extract was stored at −20 °C until analysis.

Analysis was performed on an Agilent 6890 gas chromatograph with flame ionization detection (GC/FID) using a 15 m × 0.53 mm, 1 μm film thickness DB-WAX capillary column (Agilent). Under constant flow conditions of 3 mL/min helium, a 1 μL splitless injection was performed. The oven temperature was programmed as follows: initial temperature of 65 °C for 2 min; 4 °C/min to 85 °C, 2 min hold; 20 °C/min to 235 °C, 2 min hold; 18.5 min total GC runtime.

The GC/FID was calibrated for l-menthol (CAS # 216-51-5, Acros) and (−)-nicotine (CAS # 54-11-5, Sigma–Aldrich) using seven calibration standards prepared in extraction solvent and ranging in concentration from 5 to 1000 μg/mL. The calibration was verified by analysis of a secondary source calibration verification standard prepared using menthol and nicotine obtained from sources (Sigma–Aldrich and ChemService, respectively) other than those used to prepare the primary calibration standards. Quality control samples were prepared and analyzed along with each batch of cigarettes extracted. These quality control samples consisted of continuing calibration verification standards, an extraction solvent blank, an aliquot of extraction solvent spiked with known amounts of menthol and nicotine, and matrix blanks and spikes prepared using “nicotine-free” (Quest 3^®^) nonmenthol cigarettes. To generate the matrix spikes, approximately 7 mg/g and 25 mg/g of menthol and nicotine, respectively, were added to the denicotinized cigarettes, with roughly 60% and 40% of the menthol applied to the tobacco rod and filter, respectively, and approximately 95% and 5% of the nicotine added to the rod and filter, respectively. Extraction efficiencies were determined by comparison of measured amounts to nominal spiked amounts.

To qualify the extraction and analysis technique, the menthol and nicotine content of three brands of popular, commercially available menthol cigarettes (Salem FF king, Kool FF king, and Marlboro Gold FF king) and one brand of a nonmenthol cigarette (Camel FF king) were determined, along with the distributions of menthol and nicotine between the tobacco rod and filter. To verify GC/FID peak identification and to ascertain whether there were interferences in the analysis that might require the use of a more sophisticated analytical technique, these analyses were also performed by GC with detection by mass spectrometry (MS) using the identical temperature program with a similar column (30 m × 0.32 mm, 0.50 μm film DB-WAX [Agilent]), similar constant flow rate (2 mL/min helium), a 15:1 split 1 μL injection, and full scan over the mass range 35–300 amu.

### 2.2 Cigarette mentholation

A popular, commercially available nonmenthol cigarette (Camel full flavor [FF], hard pack, king [85 mm length]), was selected for mentholation as it matched the tar, nicotine, and ventilation levels of a popular menthol brand. Cigarettes were purchased commercially and stored before use at approximately 4 °C in their sealed original packages. Prior to mentholation, 200 cigarettes (one carton) were conditioned for at least 48 h at 22 ± 1 °C and 60 ± 3% relative humidity in clean glass baking dishes [31].

Menthol crystals (l-menthol, Sigma–Aldrich) were pulverized and manually sieved using #12- and #30-size sieves (U.S. Standard Test Sieves, Advantech Manufacturing, New Berlin, WI) to generate menthol crystals with the largest dimension nominally ranging between 0.6 and 1.7 mm. The sieved crystals (500 ± 5 g) were placed into a stainless steel pan with a wire rack (rack dimensions 41 cm × 22 cm) so that 100 of the conditioned cigarettes were in a single layer and elevated 4 cm above the bed of pulverized menthol. The assembled mentholation chamber containing the cigarettes was sealed in a large resealable plastic bag and placed in a temperature- and relative humidity-controlled (30 ± 2 °C; 32 ± 5%) environment. An identical chamber containing the remaining 100 conditioned cigarettes, but without menthol crystals, served as the control. Once vapor deposition was completed, cigarettes from the mentholation and control groups were stored separately at room temperature, in two resealable plastic bags placed into a food-grade resealable plastic container.

An initial experiment was conducted to determine the rate of mentholation with respect to time. Following commencement of menthol vapor deposition, cigarettes from the mentholation and control chambers were randomly selected for analysis of menthol and nicotine content of the combined tobacco rod and filter every 24 h for a duration of 96 h. A series of experiments were subsequently performed to evaluate and qualify the custom mentholation procedure to demonstrate that the mentholated cigarettes differed only in menthol content. These experiments included an evaluation of the reproducibility of the procedure; an assessment of the effect of the mentholation process, if any, on the cigarette's nicotine content; measurement of the distribution between the tobacco rod and filter of the menthol and nicotine content in the custom-mentholated cigarettes; determination of the loss of the vapor-deposited menthol over time; and the measurement of the transfer efficiency of menthol and nicotine to mainstream smoke.

Five batches of 100 cigarettes were mentholated for 72 h each at different times over the course of two months. Five mentholated and five control cigarettes from each batch were extracted immediately (within approximately 2 h) upon completion of the 72-h vapor deposition period. The menthol and nicotine content of both the tobacco rod and filter were subsequently determined. These measurements informed the reproducibility of the custom mentholation procedure and the distribution of menthol and nicotine in the rod and filter of the custom-mentholated cigarettes, and allowed for the determination of the effect of mentholation, if any, on nicotine content.

To investigate the loss of menthol and nicotine from stored custom-mentholated cigarettes over time, we analyzed the menthol and nicotine content of cigarettes from 10 discrete batches mentholated at different times over a period of 11 months. On a specific day for a given batch, we randomly selected sample sets of five mentholated and three control cigarettes. To start, a sample set was collected and extracted immediately following completion of the 72-h vapor deposition period. Following this, three to six additional sets of cigarettes were collected from each batch on a specific day (typically 7–10 days apart) over the 35-day storage period. The tobacco in the rod of each cigarette was extracted and analyzed for menthol and nicotine content.

To determine transfer efficiencies of menthol and nicotine from the tobacco to mainstream smoke, we combined results from the analyses of the menthol and nicotine content in the unburned custom-mentholated cigarettes with corresponding measurements of menthol and nicotine in the total particulate matter (TPM) from the mainstream smoke obtained by machine smoking the cigarettes. A linear five-port smoking machine (Hawktech FP2000, Tri-City Machine Works, USA), described in more detail elsewhere [26], [32], was used to generate the mainstream smoke from the custom-mentholated cigarettes according to the International Organization of Standards/Federal Trade Commission (ISO/FTC) protocol (35 mL puff volume, 2 s puff duration, and one puff every 60 s for each cigarette). Briefly, four TPM samples were collected (one per cigarette) by sequentially smoking four randomly selected custom-mentholated cigarettes from the same batch for seven puffs per cigarette. Experiments were performed with the custom-mentholated cigarettes immediately following the completion of the 72-h mentholation period. TPM was collected on a 44-mm quartz fiber filter pad for further analysis. The TPM mass was estimated from the difference in the weight of the filter pad before and after mainstream smoke collection using a microbalance. Individual TPM filters were extracted for analysis of menthol and nicotine based on procedures previously developed for similar chemicals and matrices [26], [32], [33], [34]. The samples were extracted with 50% dichloromethane in acetonitrile and subjected to additional cleanup, as necessary, using solid phase extraction. The extracts were analyzed by gas chromatography/mass spectrometry (GC/MS) [33], [35].

## 3 Results

Before mentholation experiments could begin, it was necessary to develop and demonstrate the validity of a method for the extraction and analysis of both menthol and nicotine from the tobacco rod and cigarette filter. We present these results first, then those of the custom mentholation technique.

### 3.1 Cigarette menthol and nicotine content

Instrument calibration response was linear over the selected concentration range, such that the concentrations of primary and secondary source calibration verification standards always back-calculated to be within 12% of expected values. Solvent blank results were typically below the lower limit of quantitation of 5 μg/mL (corresponding to less than approximately 0.17 mg/g) for both menthol and nicotine. Menthol was usually not measured above 5 μg/mL in matrix blanks, yet nicotine was consistently detected in the matrix blank at approximately 50 μg/mL, corresponding to a nicotine concentration of approximately 1.7 mg/g. This is consistent with the published nicotine level of reformulated Quest 3 cigarettes of 1.0 mg/cigarette, which is roughly equal to 1.7 mg/g [36], where the conversion takes into account the typical approximate mass of tobacco filler in Quest 3 cigarettes (600 mg). Known amounts of menthol and nicotine spiked onto the rod and filter of Quest 3 cigarettes were recovered well: on average, across the analysis of five different batches of custom-mentholated cigarettes, menthol was extracted with approximately 95% and 85% efficiency from rod and filter, respectively, while nicotine showed somewhat higher extraction efficiencies with approximately 93% and 97% recovered from the rod and filter, respectively.

Results from the extraction and analysis of the combined rod and filter for four brands of commercial cigarettes using the method developed for this study are shown in Table 1. Menthol results compare quite well with those given by Celebucki et al. [37] and in the recent Food and Drug Administration/Tobacco Products Scientific Advisory Committee report ([38], p. 18), where the latter references tobacco manufacturers’ claims that characterizing levels of menthol are achieved at 1.2 mg/g menthol and that most menthol cigarettes contain at least 3 mg/g menthol. Nicotine results are consistent with those for cigarette tobacco filler previously reported [39], [40]. The distributions of menthol between rod and filter are similar to 79% and 21%, respectively, reported by Brozinski et al. [41] for commercial menthol cigarettes. To the best of our knowledge, this is the first report of the distribution of nicotine between rod and filter for commercial mentholated and nonmentholated cigarettes. The fact that most of the nicotine is contained in the tobacco rod is consistent with tobacco being the source of nicotine, and the minimal transfer of nicotine from rod to filter is due to the nicotine's low volatility (vapor pressure of 0.03 mm Hg at 25 °C).

**Table 1:** Menthol and nicotine content of commercial cigarettes (tobacco rod and filter combined) analyzed by GC/FID and GC/MS (mg/g tobacco, mean ± standard deviation, *n* = 3) and compared to reported values. Distributions of menthol and nicotine between rod and filter are also shown as determined by both analytical methods.

Cigarette brand	Menthol						Nicotine					
	GC/FID	GC/MS	%RPD^a^	Reported value^b^	GC/FID %Rod/%Filter	GC/MS %Rod/%Filter	GC/FID	GC/MS	%RPD	Reported value^c^	GC/FID %Rod/%Filter	GC/MS %Rod/%Filter
Salem FF king	2.57 ± 0.17	2.46 ± 0.17	4.3	2.60	77.0/23.0	76.5/23.5	17.8 ± 0.5	16.5 ± 0.3	7.8	18.6 ± 0.5	99.7/0.3	99.7/0.3
Kool FF king	3.14 ± 0.12	3.03 ± 0.12	3.5	3.58 ± 0.04	86.9/13.1	86.6/13.4	19.5 ± 0.7	18.6 ± 1.3	4.8	18.6 ± 0.5	99.8/0.2	99.7/0.3
Marlboro menthol gold FF king	4.55 ± 0.05	4.34 ± 0.03	4.7	2.83 ± 0.13	68.1/31.9	67.7/32.3	18.6 ± 0.3	17.4 ± 0.2	6.8	18.6 ± 0.5	99.6/0.4	99.6/0.4
Camel king (nonmenthol)	<0.17^d^	<0.17	NA^e^	NA	NA	NA	17.8 ± 0.4	17.2 ± 1.0	3.7	18.8 ± 0.6	99.6/0.4	99.6/0.4

a RPD calculated as [|GC/MS result − GC/FID result|/(average result)] × 100.

b See Celebucki et al. [37].

c See WHO [40]. Benowitz [39] reports a concentration range of 13.3 and 26.9 mg/g, without specification regarding cigarette mentholation.

d Analytical results less than the lower limit of quantification for the analytical method (5 μg/mL ≈ 0.17 mg/g, assuming 20 mL extraction volume and 600 mg weight of tobacco filler).

e Not applicable.

Analyses conducted by GC/MS on the same extracts confirmed the levels of menthol, nicotine, and quinoline found using GC/FID and showed no interferences in the chromatogram at the retention times corresponding to these analytes. These results, taken together with the acceptable spike recoveries of menthol and nicotine and agreement with previously published measurements of menthol and nicotine in the cigarette filter and tobacco rod, effectively qualify our extraction and GC/FID analysis method as both accurate and precise for the determination of the menthol and nicotine content of unburned cigarettes.

### 3.2 Custom mentholation of cigarettes

**Fig. 1:**
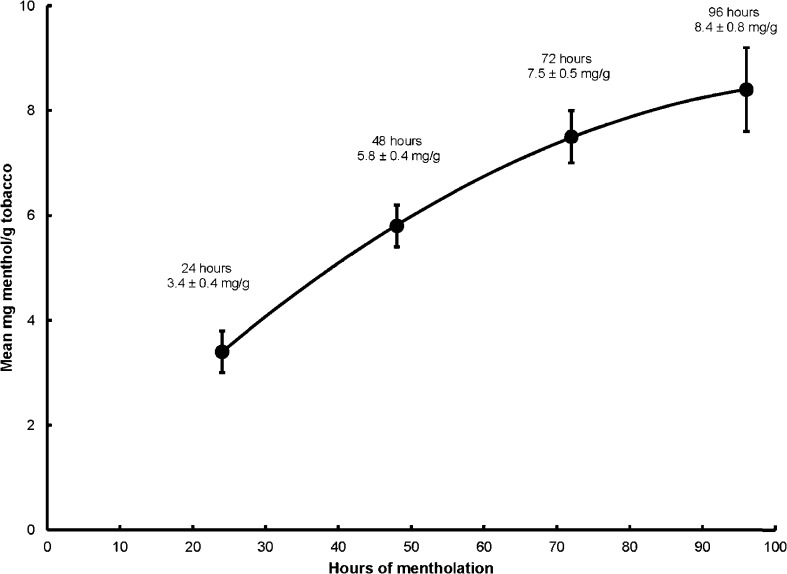
Menthol deposition as evidenced by menthol content measured over time during the custom mentholation process (mean ± standard deviation, *n* = 5 cigarettes at each time point, total cigarette menthol content of the filter and tobacco rod combined as measured by GC/FID).

We evaluated the levels of menthol in cigarettes collected after 24, 48, 72, and 96 h of custom mentholation. As anticipated, with increasing exposure of the cigarettes to the menthol crystals in the vapor deposition process, the level of menthol in the cigarettes increased, as shown in Fig. 1. Menthol was not detected above the instrumental limit of quantitation (approximately 0.17 mg/g) in any of the control cigarettes (evaluated at the same time points). This range-finding experiment showed that under the conditions selected, the menthol level ranged from 3.4 mg/g to 8.4 mg/g during the 96-h mentholation process, and the desired level of mentholation – approximately 7 mg/g, which is toward the upper end of the range of menthol content typically found in commercial cigarettes ([37], [42], pp. 22–24) – could be readily achieved following about 72 h of vapor deposition.

Menthol and nicotine levels found in the five replicate custom mentholation trials, measured each time within 2 h after 72 h of mentholation, are shown in Table 2. The average menthol and nicotine concentrations in the filter and tobacco rod combined were 6.7 ± 1.0 and 17.7 ± 0.7 mg/g tobacco, respectively, across the five trials. The desired menthol content of approximately 7 mg/g was consistently achieved in most experiments after 72 h in the mentholation chamber and the nicotine content was consistent with commercial cigarettes ([37], [40], [42], pp. 22–24). In addition, the measured difference (0.04 mg/g) between the groups of custom-mentholated and the control cigarettes is negligible and not statistically significant (*p* = 0.866).

**Table 2:** Total menthol and nicotine levels in the tobacco rod and filter of custom-mentholated and control cigarettes as measured by GC/FID (mg/g tobacco mean ± standard deviation). Distributions of menthol and nicotine between rod and filter are also shown.

Cigarette type	Experiment identifier	Menthol			Nicotine		
		Rod + filter	%Rod	%Filter	Rod + filter	%Rod	%Filter
Custom-mentholated^a^	I	7.47 ± 0.22	89.9	10.1	17.5 ± 0.6	99.6	0.4
	II	7.10 ± 0.68	91.4	8.6	16.8 ± 0.7	99.6	0.4
	III	5.24 ± 0.55	90.8	9.2	17.6 ± 0.3	99.7	0.3
	IV	6.64 ± 0.60	90.7	9.3	18.3 ± 0.4	99.7	0.3
	V	6.90 ± 0.83	91.6	8.4	18.1 ± 0.4	99.6	0.4
	Composite (*n* = 25)	6.67 ± 0.96	90.9	9.1	17.65 + 0.7	99.6	0.4

Control (nonmentholated)^b^	I^c^	<0.17^d^	Not applicable		18.3	99.6	0.4
	II	<0.17			16.5 ± 0.6	99.6	0.4
	III	<0.17			18.0 ± 0.6	99.6	0.4
	IV	<0.17			17.8 ± 0.2	99.6	0.4
	V	<0.17			18.1 ± 0.3	99.6	0.4
	Composite (*n* = 14)	<0.17			17.69 + 0.8	99.6	0.4

a *n* = 5 per experiment.

b *n* = 3 per experiment, except where noted.

c *n* = 2.

d Analytical results less than the lower limit of quantification for the analytical method (5 μg/mL ≈ 0.17 mg/g).

An examination of the results of the five separate mentholation trials shows that the menthol was deposited primarily onto the tobacco rod (91%), with a small percentage in the filter (9%). Our procedure results in a higher deposition in the rod and less in the filter, compared with the 79% and 21% for rod and filter, respectively, reported by Brozinski et al. [41] for commercial menthol cigarettes. This difference is likely due to differences in the methods used to apply the menthol to the cigarette. The distribution of nicotine between rod and filter was unchanged by the mentholation process and is consistent with other commercial brands.

### 3.3 Transfer efficiency and loss rate of nicotine and menthol

Transfer efficiencies, i.e., the ratios of menthol and nicotine in the mainstream smoke to the menthol and nicotine in the custom-mentholated cigarettes, amounted to 30% for menthol and 9% for nicotine (*n* = 3). Although our value for menthol agrees well with the 29% transfer obtained by Brozinski et al. [41], more recently reported transfer efficiencies for menthol average 10–20% ([42], pp. 22–24). Our measured value for nicotine transfer agrees well with the 10% value reported by Rodgman and Perfetti [43].

**Fig. 2:**
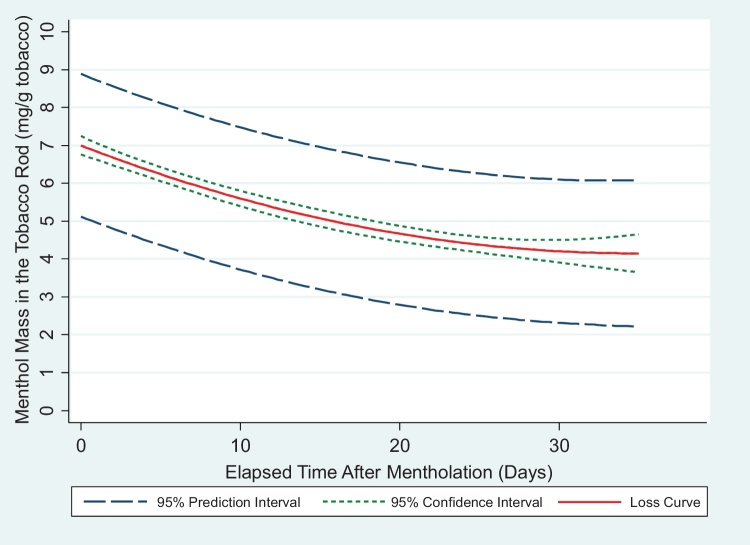
Loss of menthol over time from custom-mentholated cigarettes stored at room temperature in resealable bags contained in resealable plastic containers. Menthol = −0.1634 Day + 0.0023 Day^2^ + 7.0025 (*R*^2^ = 0.57). Menthol was measured by GC/FID.

Results for the loss rate of menthol from our custom-mentholated cigarettes, once they were removed from the vapor deposition chamber and stored, are presented in Fig. 2 as a composite plot derived from analyses of 10 discrete batches of cigarettes whose tobacco rod menthol content was measured at various times over 35 days. We fitted the menthol data as a function of time by means of a polynomial regression with both linear and quadratic terms. The amount of menthol in the tobacco rod decreased by about one-third over the first 21 days of storage, after which levels remained relatively constant. Menthol was not detected in the corresponding control cigarettes. Unlike menthol, there was no statistically significant relationship between nicotine levels and time, as nicotine content remained essentially unchanged in the custom-mentholated cigarettes over the same storage period. The data supporting these findings are provided in the Supplemental Information section.

The observed loss of menthol over time is not unexpected given its volatility (vapor pressure of 0.8 mm Hg at 20 °C, where volatile organic compounds are classified as having vapor pressures between 0.1 and 380 mm Hg). Fig. 2 shows both the 95% confidence intervals (bounding the interval within which the true value of the population mean will be found 95% of the time) and 95% prediction intervals (bounding the interval within which another single data point will be found 95% of the time). Based on these data, predicted levels of menthol in cigarettes prepared using our vapor deposition method are unlikely to be more accurate than ±2 mg/g. As a result, to ensure that the actual menthol content is known with sufficient accuracy for use in our human exposure research, we have adopted the practice of measuring the menthol content of each batch of custom-mentholated cigarettes during the calendar week in which they are smoked by subjects.

We also observe that menthol is more rapidly lost from the research cigarettes during the first ∼7 days after vapor deposition has been completed, at which point the rate of loss decreases. Because of this, in addition to determining menthol concentrations in the research cigarettes during their week of use, we do not begin using the cigarettes in our exposure studies until 7 days after the mentholation process has ended.

## 4 Discussion

We have developed extraction, analysis, and custom mentholation procedures that provide an effective means of preparing and characterizing cigarettes in which only the concentration of menthol is altered and all other constituents and design features remain unchanged. This work is an extension of our earlier effort to develop and characterize small quantities of custom-mentholated cigarettes for use in laboratory studies of cigarette smoking behavior and biomarkers of exposure [32]. We deliberately chose to generate cigarettes with menthol content somewhat higher than the average of typical commercial cigarettes so that, in related human exposure studies underway in our laboratory, we maximize the likelihood of measuring potential differences, due to the presence of menthol, in exposures to particulate matter and HPHCs. Similarly, such cigarettes could also be employed to isolate the potential effects of menthol on the toxicity of tobacco smoke.

The ability to prepare these custom-mentholated cigarettes for research purposes supplements the commercially available, dual purpose cigarette that converts from a nonmenthol to a menthol cigarette through the release of a menthol solution encapsulated in a pellet contained within the filter [32], [44]. In the custom-mentholated cigarette, the menthol is distributed between the tobacco, filter, and paper, whereas in the commercial dual purpose cigarette, the menthol is confined to the filter. Our custom-mentholation technique also offers the advantage that the menthol level can be selected and controlled.

While other methods exist for preparing mentholated cigarettes, such as application of aerosolized menthol in an alcoholic solution ([42], p. 14), we selected a vapor deposition method because of its relative ease and reasonable cost to implement on a small scale in a laboratory. In both cases (i.e., our approach and the commercial dual purpose cigarette), researchers can readily isolate the effects of menthol on smoking behavior and exposure. Work currently underway in our laboratory will determine if these menthol distributional differences between the two cigarette configurations have an effect on human smoking behavior and on exposure to particles and HPHCs in mainstream smoke.

Apart from demonstrating that the vapor deposition technique we developed was able to mentholate a nonmenthol cigarette at a selected concentration, we also showed that the procedure was predictable and repeatable, did not affect cigarette nicotine levels, and produced cigarettes in which the distribution between filter and tobacco rod was reasonably consistent for menthol and quite consistent for nicotine, and typical of commercially-available cigarettes. Transfer efficiencies of menthol and nicotine from the unburned cigarette to mainstream smoke were also similar to those reported for commercial brands. Furthermore, our previous report [32] showed that various target volatile and semivolatile HPHCs in the smoke remain essentially unchanged following cigarette mentholation.

Although the decay rate for cigarette menthol content was found to vary over time, this was not unexpected and may be accounted for by determining menthol levels in the cigarettes during the calendar week in which the cigarettes are smoked by subjects taking part in exposure studies. Furthermore, in our ongoing human exposure studies in which the custom-mentholated cigarettes have been used by numerous established smokers, no negative comments have been expressed about the research cigarettes’ acceptability with respect to either the taste or flavor of the smoke.

## 5 Conclusions

This work has important implications for future research designed to isolate the effect of menthol in cigarettes and investigate its potential role in tobacco-related disease. The development of this custom-mentholation procedure to produce cigarettes with user-defined menthol levels for controlled exposure measurements in the laboratory will allow researchers to determine if differences in smoking patterns, smoke emissions, biomarkers of exposure, and uptake of select toxins/carcinogens are attributable to the presence of menthol alone.

## Funding

This work was supported by the National Cancer Institute, National Institutes of Health (R01 CA162085 to S.S.B.). The funding agency had no involvement in the study design, in the collection and analysis of the data, nor in the preparation of this manuscript.

## Conflict of interest

The authors declare that they have no conflicts of interest.

## Transparency document

Transparency document
