# Supplementary material for: Custom mentholation of commercial cigarettes for research purposes
Source: Toxicol Rep. 2014 Oct 22;1:1068–75. doi: 10.1016/j.toxrep.2014.10.009 (PMC4303566; doi:10.1016/j.toxrep.2014.10.009)
Supplement: Supplementary file 1 [file mmc1.docx]

Table S1. Amounts of menthol and nicotine in the tobacco rod of 10 different batches of custom-mentholated cigarettes over time (mg/g tobacco).

batch	day	menthol	nicotine		batch	day	menthol	nicotine
1	0	5.52	18.44		2	12	4.61	16.70
1	0	5.16	17.35		2	21	3.43	17.33
1	0	6.83	18.22		2	21	3.55	16.17
1	0	6.52	18.38		2	21	3.62	16.75
1	0	6.42	18.07		2	21	3.38	16.62
1	1	5.40	16.53		2	21	3.68	16.85
1	1	5.79	17.53		2	26	3.43	16.07
1	1	5.63	17.99		2	26	3.59	16.97
1	1	5.46	17.83		2	26	3.60	15.93
1	1	5.45	16.46		2	26	3.59	17.10
1	4	5.77	18.13		2	26	3.48	16.26
1	4	5.37	17.77		3	0	5.85	16.27
1	4	5.35	17.12		3	0	6.91	16.21
1	4	5.38	17.09		3	0	5.84	17.32
1	4	6.15	17.31		3	0	6.47	17.00
1	7	5.98	18.51		3	15	3.61	17.63
1	7	5.09	17.64		3	15	4.50	16.32
1	7	4.46	17.99		3	15	4.05	16.90
1	7	4.67	16.64		3	15	4.17	16.52
1	7	4.72	16.57		3	15	4.69	16.93
1	13	4.98	17.75		3	22	3.19	16.04
1	13	5.31	17.77		3	22	3.97	16.95
1	13	5.15	17.37		3	22	3.45	16.10
1	13	5.22	17.79		3	22	3.33	17.39
1	13	5.22	15.02		3	22	3.42	16.63
1	28	3.85	16.78		3	32	3.28	15.91
1	28	3.90	18.29		3	32	3.25	14.78
1	28	4.16	16.10		3	32	3.31	15.97
1	28	4.60	15.44		3	32	3.39	17.38
1	28	4.51	17.34		3	32	3.44	16.24
1	35	4.09	16.53		4	0	7.48	16.15
1	35	4.04	16.77		4	0	6.09	15.97
1	35	3.62	15.80		4	0	6.89	17.01
1	35	4.12	16.76		4	0	6.93	15.88
1	35	4.37	16.37		4	0	6.88	16.36
2	0	5.59	17.50		4	8	5.66	15.91
2	0	6.05	17.74		4	8	5.72	16.35
2	0	5.83	17.70		4	8	5.47	15.41
2	0	5.90	16.76		4	8	5.76	16.43
2	0	6.01	17.64		4	8	5.48	15.80

Table S1 (continued). Amounts of menthol and nicotine in the tobacco rod of 10 differen
batches of custom-mentholated cigarettes over time (mg/g tobacco).

batch	day	menthol	nicotine	batch	day	menthol	nicotine
4	15	5.09	16.75	7	13	6.99	18.34
4	15	5.06	17.03	7	13	6.77	18.06
4	21	4.97	15.46	8	0	7.26	17.77
4	21	5.09	16.32	8	0	7.30	17.43
4	21	5.18	15.24	8	0	7.07	17.45
4	21	5.16	15.54	8	0	7.61	16.90
4	21	5.17	14.98	8	0	7.72	16.95
4	32	5.37	16.92	8	13	4.51	16.86
4	32	5.14	17.43	8	13	5.49	16.42
4	32	5.02	16.92	8	13	5.00	17.17
4	32	5.02	16.85	8	13	5.03	17.22
4	32	5.42	17.30	8	13	4.96	17.08
5	0	7.41	14.73	8	21	4.71	17.01
5	0	8.80	17.10	8	21	5.21	16.75
5	0	8.70	15.77	8	21	5.07	16.32
5	0	8.03	16.08	8	21	4.84	17.94
5	0	7.99	15.67	8	21	4.83	17.69
5	12	7.59	16.14	8	26	4.43	16.80
5	12	7.73	15.83	8	26	4.59	16.84
5	12	7.40	15.86	8	26	3.83	15.76
5	12	7.73	16.05	8	26	4.09	16.18
5	12	7.28	16.02	8	26	4.53	17.21
6	0	8.75	17.35	9	0	7.64	16.30
6	0	8.48	17.61	9	0	7.99	16.61
6	0	7.59	17.61	9	0	7.38	16.85
6	0	8.25	16.68	9	0	8.05	16.33
6	0	7.68	17.69	9	0	7.83	16.29
6	18	5.44	17.94	9	21	4.98	17.05
6	18	4.95	17.60	9	21	5.37	18.07
6	18	5.20	17.73	9	21	6.03	17.34
6	18	5.83	17.92	9	21	6.52	16.71
6	18	5.65	18.62	9	21	5.17	17.57
6	26	5.68	17.61	10	0	6.45	17.13
6	26	4.48	17.71	10	0	6.78	17.06
6	26	4.79	18.31	10	0	7.05	18.21
6	26	5.22	18.22	10	0	7.08	17.99
6	26	5.53	18.48	10	0	6.63	17.31
7	0	8.18	17.25	10	21	4.15	17.86
7	0	8.53	16.75	10	21	3.94	17.70
7	0	8.74	17.94	10	21	5.12	16.68
[illegible]	[illegible]	[illegible]	[illegible]	[illegible]	[illegible]	[illegible]	[illegible]
